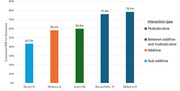# Combined population attributable fractions for dementia are dependent on strong assumptions of the form of interactions between risk factors: an analysis of data from the Canadian Longitudinal Study on Aging

**DOI:** 10.1002/alz.087708

**Published:** 2025-01-09

**Authors:** Aaron Jones, Yasaman Dolatshahi

**Affiliations:** ^1^ McMaster University, Hamilton, ON Canada

## Abstract

**Background:**

Several recent studies have used communality weights to calculate a combined population attributable fraction (PAF) of modifiable risks factors for dementia. Other research has suggested this method may be underestimating the true PAF. We applied multiple methods to calculate the combined PAF of 12 modifiable risk factors using record‐level, national Canadian data.

**Method:**

We used baseline data (2011‐2015) from the comprehensive cohort of the Canadian Longitudinal Study on Aging (CLSA). We measured the 12 risk factors identified in the Lancet Commission on dementia prevention, intervention, and care. We calculated a combined PAF of these risk factors using the independent multiplicative method of Barnes and Yaffe (BY‐M), the additive and multiplicative methods of Welberry (W‐A and W‐M), and communality‐adjusted multiplicative method of Norton (N‐M). We also implemented a novel method (J‐MA) to mix multiplicative and additive interactivity based on the number of risk factors present. All methods incorporated sample weights and used the risk ratios from the Lancet Commission.

**Result:**

There were 30,097 baseline participants in the CLSA ranging in age from 45 to 85 years (median 62). Combined PAFs varied widely: 78.5%(W‐M), 75.9% (BY‐M), 59.8% (J‐MA), 58.2% (W‐A), and 43.3% (N‐M). The BY‐M and W‐M methods resulted in unrealistically high combined risk ratios (RR >90) in individuals with many risk factors. The other methods produced broadly realistic results. Despite a nominally multiplicative form, N‐M produced results consistent with sub‐additive interactivity and makes arbitrary analytic choices. The J‐MA method also uses an arbitrary function to blend multiplicative and additive interactions. The W‐A approach made no assumption other than additive interactivity.

**Conclusion:**

Assumptions regarding how risk factors for dementia interact highly influence combined PAFs. All multiplicative methods produce either unrealistic results or require arbitrary analytic choices. The additive method makes no arbitrary assumptions but may not reflect the actual form of interactions between risk factors. Not enough is known on how modifiable risk factors for dementia interact to estimate combined PAFs with confidence. When reporting PAFs for dementia a range of possible values, including one assuming additive interactions, should be provided and the assumptions behind each value clearly stated.